# Benchmark of Data Processing Methods and Machine Learning Models for Gut Microbiome-Based Diagnosis of Inflammatory Bowel Disease

**DOI:** 10.3389/fgene.2022.784397

**Published:** 2022-02-14

**Authors:** Ryszard Kubinski, Jean-Yves Djamen-Kepaou, Timur Zhanabaev, Alex Hernandez-Garcia, Stefan Bauer, Falk Hildebrand, Tamas Korcsmaros, Sani Karam, Prévost Jantchou, Kamran Kafi, Ryan D. Martin

**Affiliations:** ^1^ Phyla Technologies Inc, Montréal, QC, Canada; ^2^ Mila, Quebec Artificial Intelligence Institute, University of Montreal, Montréal, QC, Canada; ^3^ Max Planck Institute for Intelligent Systems, Tübingen, Germany; ^4^ Gut Microbes and Health, Quadram Institute Bioscience, Norwich, United Kingdom; ^5^ Earlham Institute, Norwich, United Kingdom; ^6^ Centre Hospitalier Universitaire Sainte-Justine, Montréal, QC, Canada

**Keywords:** inflammatory bowel disease, machine learning, gut microbiome, batch effect reduction, data normalization, QIIME2, PICRUSt2

## Abstract

Patients with inflammatory bowel disease (IBD) wait months and undergo numerous invasive procedures between the initial appearance of symptoms and receiving a diagnosis. In order to reduce time until diagnosis and improve patient wellbeing, machine learning algorithms capable of diagnosing IBD from the gut microbiome’s composition are currently being explored. To date, these models have had limited clinical application due to decreased performance when applied to a new cohort of patient samples. Various methods have been developed to analyze microbiome data which may improve the generalizability of machine learning IBD diagnostic tests. With an abundance of methods, there is a need to benchmark the performance and generalizability of various machine learning pipelines (from data processing to training a machine learning model) for microbiome-based IBD diagnostic tools. We collected fifteen 16S rRNA microbiome datasets (7,707 samples) from North America to benchmark combinations of gut microbiome features, data normalization and transformation methods, batch effect correction methods, and machine learning models. Pipeline generalizability to new cohorts of patients was evaluated with two binary classification metrics following leave-one-dataset-out cross (LODO) validation, where all samples from one study were left out of the training set and tested upon. We demonstrate that taxonomic features processed with a compositional transformation method and batch effect correction with the naive zero-centering method attain the best classification performance. In addition, machine learning models that identify non-linear decision boundaries between labels are more generalizable than those that are linearly constrained. Lastly, we illustrate the importance of generating a curated training dataset to ensure similar performance across patient demographics. These findings will help improve the generalizability of machine learning models as we move towards non-invasive diagnostic and disease management tools for patients with IBD.

## Introduction

The human gut microbiome is a collection of microbes, viruses and fungi residing throughout the digestive tract. The gut microbiota plays an important role in human health, influencing food digestion, the immune system, mental health, and numerous other functions (reviewed in Mohajeri et al., 2018). Alterations in the gut microbiome have been linked to illnesses such as multiple sclerosis, type II diabetes, and inflammatory bowel disease (IBD) (Gevers et al., 2014; Opazo et al., 2018). IBD comprises two main subtypes: Crohn’s disease (CD) and ulcerative colitis (UC), characterized by periodic inflammation throughout the gastrointestinal tract or localized to the colon, respectively (Caruso et al., 2020). The prevalence of IBD is increasing globally over the last several decades, from 79.5 to 84.3 per 100,000 people between 1990 and 2017, with Canada having among the highest IBD rates at 700 per 100,000 people in 2018 (Benchimol et al., 2019; GBD 2017 Inflammatory Bowel Disease Collaborators, 2020). Although the disease etiology is currently undetermined, the increasing rates of IBD have been linked to lifestyle factors, such as a Western diet (Rizzello et al., 2019).

Currently, IBD diagnosis and monitoring is primarily performed via blood tests, fecal calprotectin, and endoscopies which can be costly, invasive, and display variable accuracy, all of which leads to delayed diagnosis and infrequent disease monitoring (Ricciuto et al., 2021) (Ricciuto et al., 2021). Therefore, there is an unmet need for the development of further non-invasive, low-cost, and rapid methods for screening, diagnosis, and disease management for the growing number of IBD patients (Armstrong et al., 2008; Noiseux et al., 2019). One potential diagnostic test within these constraints involves using the gut microbiome composition to identify patients with IBD.

Over the past decade, several studies have compared the gut microbiome profiles of healthy individuals and those with CD or UC (McHardy et al., 2013; Gevers et al., 2014; Walters et al., 2014; Liu et al., 2016; Tedjo et al., 2016; Duvallet et al., 2017; Halfvarson et al., 2017; Pascal et al., 2017; de Meij et al., 2018; Vázquez-Baeza et al., 2018; Pittayanon et al., 2020; Clooney et al., 2021). Common characteristics of the gut microbiome identified in patients with IBD are the reduction in bacterial diversity and development of a dysbiotic state, referring to alterations in the structure and function of the gut microbiome compared to healthy individuals (McHardy et al., 2013; Duvallet et al., 2017; Pascal et al., 2017). Principal coordinate analysis with UniFrac (Halfvarson et al., 2017) or Bray-Curtis (Clooney et al., 2021) distance of the gut microbiome’s composition has identified differential clustering of healthy and IBD samples. Although the dysbiotic state is commonly identified in IBD patients, it remains unknown whether the microbiome initiates IBD or is only a reflection of the patient’s current health status. Larger meta-analyses have aimed to identify differentially abundant taxa between IBD patients and healthy controls in order to generate potential diagnostic biomarkers, although with limited success to date (Walters et al., 2014).

Due to difficulties identifying biomarkers with standard statistical methods for disease diagnosis, the field has moved to applying predictive machine learning (ML) models for classification of patient phenotypes. Several studies have demonstrated accurate classification of patients with IBD from their gut microbiome profile with ML models (Gevers et al., 2014; Walters et al., 2014; Tedjo et al., 2016; Ananthakrishnan et al., 2017; Duvallet et al., 2017; de Meij et al., 2018; Douglas et al., 2018; Topçuoğlu et al., 2020). Common ML models employed for IBD classification include random forest (collection of decision trees for classification) (Gevers et al., 2014; Tedjo et al., 2016), logistic regression (binary linear classifier) (de Meij et al., 2018), and neural networks (layers of differently weighted nodes contributing to a classification) (Ananthakrishnan et al., 2017; Topçuoğlu et al., 2020).

Features commonly used for IBD classification with ML models can be categorized into three groups: clinical, bacterial, and functional. Clinical features encapsulate those regarding the patient (i.e., age, sex, body mass index (BMI)) and results from other clinical tests (i.e., calprotectin, colonoscopy) (Waljee et al., 2017). Taxonomy and functional features are usually determined via sequencing-based microbiome profiling, such as amplicon sequencing of the 16S rRNA gene or whole genome shotgun (WGS) sequencing of all DNA in a sample (Berg et al., 2020). Bioinformatic tools, such as QIIME2 (Bolyen et al., 2019) or LotuS2 (Hildebrand et al., 2014), provide pipelines for clustering 16S rRNA-amplicon sequences into operational taxonomic units (OTUs) which can then be compared to public databases to find taxonomy assignments (Quast et al., 2013). WGS reads are frequently used to infer potential functions represented in the genomes of microbial community members (reviewed in Frioux et al., 2020). Similarly, we can use known genomes in public databases to derive functional predictions in a community based solely on amplicon sequencing based taxonomy profiles, implemented in tools such as PICRUSt2 (Douglas et al., 2020). Although WGS provides greater taxonomic resolution and estimates of microbiome functions, 16S rRNA amplicon sequencing is currently more applicable to a diagnostic test due to its speed, affordability, and standardization of analysis tools.

A critical, and often under-explored, consideration for generating ML models for disease classification is their generalizability to previously unseen cohorts of patients. A ML model that underperforms when presented with data from a new patient cohort is not reliable enough to be applied in a clinical setting (Ho et al., 2019). Despite this, models currently used in the context of microbiome data are often only trained and cross-validated with different splits of data from the same cohort. In studies where cross-validation with an unseen sample cohort is performed, the performance of models is often lower, indicative of the model overfitting to the training set (Ananthakrishnan et al., 2017; Douglas et al., 2018). A proposed explanation for the reduced performance is the potential for non-biological variability, commonly referred to as batch effects, introduced to the data by wet-lab protocols and sequencing instruments during the processing of these samples, typically observed in meta-analysis of microbiome data (Duvallet et al., 2017).

In order to improve model performance on unseen data, it is necessary to apply data normalization or transformation and batch effect correction techniques prior to model training. Normalization is a critical step to remove biases to feature abundance estimates, such as the data’s compositional nature, heteroskedasticity, or skewness. For example, microbiome data’s compositional nature prevents the direct application of standard statistical methods as they may lead to erroneous results, and requires prior application of compositional transformation methods (Gloor et al., 2017; Weiss et al., 2017). In addition, methods have been developed to remove technical batch effects commonly observed in collections of samples from different studies, such as naive zero-centering methods, Meta-analysis Methods with a Uniform Pipeline for Heterogeneity (MMUPHin), and ComBat-seq (Nygaard et al., 2016; Gibbons et al., 2018; Ma et al., 2020; Wang and LêCao, 2020; Zhang et al., 2020). To date, the effect of various combinations of normalization and batch effect correction techniques on ML model generalizability remains to be benchmarked.

In this article, we propose a standardized approach for evaluating the performance and generalizability of data processing pipelines and ML models with microbiome data to classify patients with IBD. Previous microbiome ML benchmarking studies focused on performance of various combinations of model type, normalization or transformation, and microbiome compositional features using variations of five-fold cross validation (Song et al., 2020; Topçuoğlu et al., 2020). Five-fold cross validation fails to assess the generalizability to new, unseen sample batches as each split potentially contains samples from all batches present in the dataset. Therefore, we implemented a leave-one-dataset-out (LODO) (Thomas et al., 2019) cross-validation method to directly assess cross-batch generalizability. In this approach, the model is iteratively trained on samples of all but one dataset and then tested on the left-out dataset. Different combinations of data types, normalization or transformation methods, batch effect correction methods, and ML models were assessed in order to establish a comprehensive performance benchmark of microbiome-based disease classification in the context of IBD.

## Methods

### Acquisition of Sample Data

Sample FASTQ files were acquired from the European Nucleotide Archive (ENA) browser. The sample metadata including covariates such as BMI, life stage, sex, IBD subtype, disease status and sample type was acquired from the corresponding publication’s supplementary materials or the QIITA microbiome platform (Gonzalez et al., 2018). Samples collected from individuals in North America and with more than 3,000 counts following processing of the raw reads (as described in methods) were retained from each dataset. The dataset ENA accessions and technical information regarding the samples in each dataset are available in Table 1.

**TABLE 1:** Overview of QIIME2 processing for 15 microbiome datasets. Samples were collected from the listed ENA accession, with only samples corresponding to individuals in North America retained. SR refers to single read and PE to paired-end sequencing runs for the corresponding length in bp. Trim length was used as input for the trunc_len parameter, forward trim as the trim_left input for single end read and trim_left_f for paired-end reads, and reverse trim as the trim_left_r input for paired-end reads in the python API for QIIME2’s Dada2 plugin.

Study ID	ENA Accession	Hypervariable Region	Trim Length	Forward Trim	Reverse Trim	Mean Reads	SD
American Gut	ERP012803	V4	124	0	0	30864.7	30259.6
CVDF	PRJNA308319	V3-V4	290	40	40	402901.1	77178.6
GEVERSC	PRJEB13680	V4	174	0	0	76323.2	62701
GEVERSM	PRJEB13679	V4	174	0	—	40903.8	41606.2
GLS	PRJEB23009	V4	99	0	—	75901.4	59442.8
HMP	ibdmdb.org	V4	249	0	0	44154.7	15479
MUC	PRJNA317429	V4	174	19	21	84848	36257.9
PRJNA418765	PRJNA418765	V4	245	0	3	24329.9	14107.2
PRJNA436359	PRJNA436359	V4	170	0	3	80925.9	90657.2
QIITA10184	PRJEB13895	V4	120	0	—	93773.8	31830.3
QIITA10342	PRJEB13619	V4	100	0	—	78873.1	68866.2
QIITA10567	PRJEB14674	V4	99	0	—	17547.5	7,522.5
QIITA1448	PRJEB13051	V4	99	0	—	159739	57806.5
QIITA2202	PRJEB6518	V4	99	0	—	243402.8	190268.4
QIITA550	PRJEB19825	V4	149	0	—	37658.8	7,065.2

The following fifteen studies were included in our dataset:

1. The American Gut cohort is from a large, open platform which collected samples from individuals in the US to identify associations between microbiomes, the environment, and individual’s phenotype (McDonald et al., 2018). We included available samples that did not contain any self-reported diseases in the metadata.
2. The CVDF study determined the effect of cardiorespiratory fitness on microbiome composition and comprises a range of fitness levels (Estaki et al., 2016; McDonald et al., 2018).
3. The GEVERSM study assessed the microbiome composition of treatment naive, newly diagnosed, paediatric patients with IBD and adult patients diagnosed with IBD for 0–57 years (Gevers et al., 2014).
4. The GEVERSC cohort consists of additional samples from paediatric and adult patients added to the GEVERSM study (Gevers et al., 2014).
5. The GLS study longitudinally sampled 19 patients with CD (Crohn’s disease activity index (CDAI) between 44 and 273) and 12 healthy control individuals (Vázquez-Baeza et al., 2018).
6. The Human Microbiome Project (HMP) study longitudinal tracked pediatric and adult patients ranging from newly diagnosed to diagnosed for 39 years. Diagnosis was confirmed by colonoscopy prior to enrollment in the study along with several other inclusion criteria listed in the corresponding publication (Vázquez-Baeza et al., 2018; Lloyd-Price et al., 2019).
7. The MUC study collected mucosal biopsies from 44 pediatric patients with CD and 62 non-IBD pediatric control patients (Liu et al., 2016).
8. PRJNA418765 was a longitudinal study of patients with CD that were refractory to anti-TNF initiating ustekinumab assessed at week 0, 4, 6 and 22. To be included, patients required at least 3 months of Crohn’s disease history and a CDAI between 220 and 450 (Doherty et al., 2018).
9. PRJNA436359 was a longitudinal study of new onset and treatment naive pediatric patients with UC receiving a variety of medications at week 0, 4, 12, and 52. Inclusion criteria consisted of presence of disease beyond the rectum, Pediatric Ulcerative Colitis Activity Index (PUCAI) of 10 or more, and no previous therapy (Schirmer et al., 2018).
10. QIITA10184 was a study comparing five different faecal collection methods and their effect on the healthy participant’s microbiome composition identified with 16S rRNA gene sequencing (Vogtmann et al., 2017).
11. QIITA10342 study assessed the microbiome composition and function of healthy individuals in two American Indian communities in the United States (Sankaranarayanan et al., 2015).
12. QIITA10567 samples consist of the control individuals in a study linking alterations in microbiome composition to Parkinson’s disease (Hill-Burns et al., 2017).
13. The QIITA1448 study compared microbiome composition of individuals in traditional agricultural societies in Peru to those in industrialized cities in the United States (Obregon-Tito et al., 2015).
14. The QIITA2202 study collected longitudinal stool samples from two healthy individuals alongside detailed lifestyle characteristics to correlate with microbiome composition (David et al., 2014).
15. The QIITA550 study collected longitudinal stool samples from two individuals to assess temporal changes in microbiome composition (Caporaso et al., 2011).

### Taxonomy Classification With QIIME2

Taxonomy abundance tables were generated from the FASTQ files using QIIME2 (v2020.2) (Bolyen et al., 2019). Only samples from the same study were processed together. Reads were trimmed to remove low quality base pairs (trimming parameters listed in Table 1), chimeras removed, and sequences denoised using Dada2 (Callahan et al., 2016) or Deblur (for GLS and AG due to technical issues of processing these samples with Dada2) (Amir et al., 2017). Closed reference OTU clustering with the Silva 132 99% reference database (Quast et al., 2013; Yilmaz et al., 2014; Glöckner et al., 2017) was performed with the cluster_features_closed_reference function from QIIME2 plugin VSEARCH (v2.7.0) (Rognes et al., 2016) at 99% similarity. The resulting centroid sequences were classified with a Naive Bayes classifier (Wang et al., 2007) at a 99% confidence cut-off. In order to train the Naive Bayes classifier, the sequences of the 16S rRNA hypervariable region sequenced in the respective study (either V3-V4 or V4) were extracted from the Silva 132 99% full length 16S OTU reference with the extract-reads function from the QIIME2 feature-classifier plugin. The extracted reads and the corresponding taxonomy labels were used to train the Naive Bayes classifier with the QIIME2 plugin feature-classifier’s fit-classifier-naive-bayes function. Resulting taxonomic feature tables were collapsed to species (level 7) and genus (level 6) classification for further analysis.

### Inferring Functional Abundance With PICRUSt2

Functional abundance tables were generated using PICRUSt2 (v2.3.0) from the OTU abundance table and representative OTU sequences generated using QIIME2. We generated abundance tables from the six different databases incorporated into PICRUSt2: Clusters of Orthologous Groups of proteins (COG), Kyoto Encyclopedia of Genes and Genomes (KEGG) orthologs (KO), Enzyme Commission (EC), Pfam protein domain (PFAM), TIGR protein family (TIGRFAM) and MetaCyc pathways. Each database is independently curated and provides information on different aspects of the functional properties present in the microbiome.

### Feature Selection

Following taxonomy classification and inference of functional abundance, features present in less than 10% of the samples within each study in the training set were pruned from the dataset. Following pruning of the training set, the test set was subset to those features as well.

### Data Normalization and Transformation Methods

When possible, normalization and transformation methods were implemented using python (v3.6.12) and R (v3.6.3) packages with the methods already incorporated. For CLR and ILR transformation, zero values were first replaced with a multiplicative replacement function that replaces zeros with a small positive value (equal to 1/N^2^, where N equals the number of columns) and ensures the sum of the row remains 1 (Martín-Fernández, 2003) prior to transformation with the clr and ilr functions, respectively, from the python package SciKit-Bio (v0.5.2). CLR performs a log transformation of abundance values, which are normalized by the geometric mean of all features. ILR uses a change of coordinate space projection calculation to transform proportional data (or relative abundances) to a new space with an orthonormal basis, in this case the J.J.Egozcue orthonormal basis (Egozcue, 2003).

For TSS normalization, the counts for each feature were divided by the sum of all feature counts in the sample with a custom python function. The method constrains the sample row sum to one, aiming to similarly scale all samples while maintaining biological information of microbial abundances. For ARS normalization, the TSS normalized values were transformed with the sqrt function followed by the arcsin function from the python package Numpy (v1.19.2). The LOG normalization was also applied to the TSS normalized values using the log function from numpy following replacement of all zero values with the multiplicative replacement function.

For VST normalization, we used the varianceStabilizingTransformation function in the R package DESeq2 (v1.26.0). VST aims to factor out the dependence of the variance in the mean abundance of a feature. The method numerically integrates the dispersion relation of the feature mean fitted with a spline, evaluating the transformation for each abundance in the feature. VST normalization was performed by normalizing the training set separately from the test set as the normalization is dependent on all samples present in the dataset.

### Batch Effect Correction Methods

We explored three methods for batch effect correction: naive zero-centering, an empirical Bayes method, and a negative binomial regression method. The naive zero-centering batch effect correction entails centering the mean of each feature within a batch to zero (Nygaard et al., 2016). We also assessed MMUPHin, an empirical Bayes method designed specifically for zero-inflated microbial abundance data. MMUPHin estimates parameters for the additive and multiplicative batch effects, using normal and inverse gamma distributions, respectively. The estimated parameters are then used to remove the batch effects from the dataset (Ma et al., 2020). MMUPHin was implemented with a custom python script. Lastly, ComBat-seq fits the feature counts to a negative binomial regression model to estimate the batch specific parameters. The batch specific parameters are used to calculate a ‘batch-free’ distribution which the raw counts are mapped to in order to obtain the final corrected data (Zhang et al., 2020). ComBat-seq was implemented with the ComBat_seq function from the R package SVA (v3.38.0) (Parker et al., 2014). We considered a batch as the whole dataset or split a dataset into multiple batches when the metadata indicated different sample preprocessing methods or samples were processed in different locations.

Two variations of MMUPHin and ComBat-seq were implemented to ensure the batch effects were corrected from the training and test sets separately. For variation #1, the test study’s samples were removed to generate the training set. Training set batch effect correction was completed, with the sample type (stool/biopsy) provided as a biological covariate, and the corrected values used for training the ML model. For the test set in variation #1, batch effect correction was performed with the full dataset and then the test study’s samples were collected to form the test set. The corrected test was then used to assess the model’s classification performance (Supplemental Figure S1, variation #1). For variation #2, batch effect correction was completed on the training set prior to model training with both the sample type and disease label (UC/CD/Control) provided as biological covariates. The model’s classification performance was then assessed on samples from the test dataset which were not corrected (Supplemental Figure S1, variation #2). Lastly, feature abundance for some samples following batch effect correction of the OTU dataset with MMUPHin were all zero. To ensure these samples were compatible with the compositional transformation methods, we set all features of these samples to equal 1/N (where n is the number of features) prior to transformation. For all other normalization methods, the feature abundance was not adjusted.

### Assessing the Mixing of Batches Following Batch Effect Correction

We assessed the ability of the three batch effect correction methods to improve mixing of samples from different batches with the beta-diversity metric Aitchison distance, equivalent to the Euclidean distance between CLR transformed microbiome data (Quinn et al., 2018), and the Local Inverse Simpson Index (LISI) (Polański et al., 2020). The genus abundance dataset was filtered to include features present in 10% of the samples from at least one batch, followed by CLR transformation and batch effect correction applied in the same manner as in our classification pipeline. Dimensionality reduction with principal component analysis (PCA) using the PCA function from the python package SciKit-Learn (v0.22.1) was performed with the resulting clr transformed and batch effect corrected values. The first two principal components were used to generate the scatter plots visualizing the separation of labels corresponding to diagnosis and sample batch. In order to visualize the different labels, a 95% confidence ellipse was added for each disease or batch label in the respective graph.

In order to quantify the mixing or separation of disease and batch labels, the LISI metric was calculated using the first 50 principal components with the compute_lisi function from the python package harmonypy (v0.0.5**)** (Korsunsky et al., 2019). LISI selects the nearest neighbors of a sample to calculate the inverse Simpson’s index for the diversity of labels surrounding the sample. For the batch integration LISI (iLISI) score, the batch label was provided and for disease LISI (dLISI) score the disease label was provided. The iLISI and the dLISI were calculated for every sample following each batch effect correction method with the first 50 principal components following dimensionality reduction with PCA. The median score for each method was determined and scaled with the overall minimum and maximum scores to a range between 0 and 1 (Tran et al., 2020). A Wilcoxon signed-rank test with Benjamini and Hochberg *p*-value correction was applied to determine if the values were significantly different. In order to assess the overall effect of the batch effect correction methods, the harmonic mean (also referred to as the F1 score) incorporating both the iLISI and dLISI was calculated as previously described (Lin et al., 2019; Tran et al., 2020).

#### Standard Machine Learning Models

We assessed the classification performance of six standard machine learning models and two deep learning models. The six standard models were implemented using the python package SciKit-Learn (v0.22.1). Hyperparameters were not optimized and decided prior to experimentation.

#### Bernoulli Naive Bayes Classifier

The Bernoulli Naive Bayes Classifier (BNB) model converts the feature space to binary values and then estimates parameters of a Bernoulli distribution for classification purposes. We implemented the BNB model using the default settings in SciKit-Learn.

#### Random Forest

Random Forest (RF) models use an ensemble of decision trees that discriminate the feature space by a sequence of threshold conditional statements. The power of the model comes from its non-linear classification capabilities and the number of trees used to label classification. We implemented the Random Forest classifier with the following modifications to the default SciKit-learn settings: n_estimaters = 500 and class_weight = balanced.

#### K-Nearest Neighbour Classifier

The K-Nearest Neighbour Classifier (KNN) classifies each sample by majority vote of the K nearest neighbours in its surrounding. We implemented the K-Nearest Neighbors classifier with the following modifications to the default SciKit-learn settings: n_neighbors = 6, weights = distance, and metric = manhattan.

#### Support Vector Machine Classifier

The Support Vector Machine Classifier (SVC) identifies multivariate decision boundaries that separate class labels. We implemented two SVC variations, the first with a linear kernel, constraining the decision boundary to a linear hyperplane, using the SGDClassifier class from SciKit-learn with the following modifications to default settings: loss = modified_huber, tol = 10e-5, and max_iter = 10,000. The second variation used the radial basis function kernel with the SVC class from SciKit-Learn, which removes the linear constraint of the decision boundary, with the following modifications to the default settings: tol = 10e-6, class_weight = balanced, and max_iter = 100000.

#### Logistic Regression

Logistic Regression classification estimates the probability of a certain class in a binary classification problem using a statistical fit to the logistic function. We implemented the LogisticRegression class from SciKit-Learn with the following modifications to the default settings: solver = sag, class_weight = balanced, and max_iter = 10,000. For the non-linear variation, the feature space was first transformed with the radial basis function kernel implemented with the rbf_kernel function from SciKit-Learn prior to fitting a logistic regression model.

#### Gradient Boosted Trees

Gradient boosted trees consist of a collection of sequential decision trees, where each tree learns and reduces the error of the previous tree (Chen and Guestrin, 2016). The gradient boosted trees model was implemented with the XGBoost package’s (v1.2.0) XGBoostClassifier class with the following modifications to default settings: n_estimators = 500.

### Deep Learning Models

The deep learning models were built with the python package Tensorflow (v2.2.0). The models were trained for up to 100 epochs with a batch size of 16 and samples shuffled. The best weights were selected using early stopping (EarlyStopping callback) by monitoring the validation loss (5% split of the training set) with a min_delta = 1 × 10^−3^ and patience = 10.

#### Multilayer Perceptron

A MLP is a neural network architecture composed of one or more layers of fully connected neurons that take as input the weights of the previous layer and output the result of an activation function to the subsequent layer. For binary classification, the final layer contains a single node that predicts the class probability. We implemented an MLP architecture with three hidden layers of 256 neurons using a rectified linear unit (ReLU) activation function followed by a Dropout layer with a dropout rate of 50%. The final layer predicted the class label with a sigmoid activation function. The model was trained using a binary cross entropy loss function and the Adam optimizer with a learning rate of 0.001.

#### Convolutional Neural Network

We implemented MDeep, a CNN architecture recently designed for microbiome data (Wang et al., 2021). CNNs require an inherent structure to be present in the data, which is added to the OTU dataset by hierarchical agglomerative clustering of the phylogeny-induced correlation between OTUs. We built a phylogenetic tree with the align_to_tree_mafft_fasttree function in the QIIME2 phylogeny python plugin using the OTU representative sequences obtained from clustering 16S rRNA sequences with QIIME2. The phylogenetic tree was imported into R using the phyloseq package and the cophenetic distance between OTUs determined with the R package ape. The cophenetic distance was then used to calculate the phylogeny-induced correlation as described in the original study and OTUs clustered using the HAC function from the MDeep GitHub repository (https://github.com/lichen-lab/MDeep).

### Leave One Dataset out Cross Validation

The generalizability of each model, normalization or transformation, and batch effect correction method, were determined through a cross validation strategy which assessed predictive performance on previously unseen batches of samples. As there were 15 studies, we iterated through the full dataset 15 times, generating the training set by removing all samples from a single study to create a separate test set. Feature selection was performed with the training set, followed by normalization and batch effect correction with the respective methods to both the training and test sets. Lastly, the number of non-IBD control and IBD samples in the training set were balanced by randomly subsampling the label with the greater number of samples, while maintaining the proportion of samples from each batch, disease label (UC/CD/Control), and sample type (stool/biopsy).

To measure the performance of the various normalization, transformation, batch effect correction, and model combinations, we calculated two metrics for binary classification: F1 score and Matthews correlation coefficient (MCC) (Wardhani et al., 2019). In the LODO cross-validation, some test sets contained only a single label thereby preventing the calculation of the MCC and F1 Score. In order to be able to include these predictions in the overall performance, the F1 Score and MCC were calculated by combining the test set predictions of all 15 folds. As the number of samples in a study ranged from 23 to 1,279, there was potential for the overall performance metrics to be skewed by the predictions of a single study with a large number of samples. Whereas in a typical 5-fold cross-validation each fold is weighted equally, with the classification performance determined separately for each fold and then an overall average calculated. Therefore, in order to calculate the pipeline’s classification performance with equal weighting to each study, the confusion matrix for each study was generated and normalized by the number of samples in the study. The average proportion of true positives, true negatives, false positives, and false negatives across the 15 studies was used to generate an overall confusion matrix. The overall F1 score and MCC were calculated from the averaged confusion matrix with the following equations.

For the pipeline combinations with the best overall classification performance, the classification accuracy was also determined for each individual dataset. The accuracy was calculated from the normalized confusion matrix with the following equation:

### Sample Subgroup Performance Analysis

We assessed the difference in classification performance of patients within five different metadata variables (age, BMI, sample type, sex, IBD type), each with two categorical labels coded as 0 or 1. The samples were grouped by the five variables and the classification performance metric within each group calculated. For the logistic regression analysis, the performance metric was input as the dependent variable and the five metadata groups as the independent variables. The MCC score was scaled with the MinMaxScaler from SciKit-Learn to a range from 0 to 1 as required for the logistic function. The logistic function was fit with the Logit function from the statsmodels (v0.11.1) python package.

### Feature Importance From XGBoost Classifier

In order to determine the importance of each taxonomy, we collected the features’ gain value from our second-best pipeline composed of CLR normalized, zero-centered, genus abundance features with an XGBoost Classifier. The gain values were collected from the trained XGBoost classifier in each LODO iteration separately.

### Taxonomy Differential Abundance

Differential taxonomy abundance was performed with Analysis of Compositions of Microbiomes with Bias Correction (ANCOM-BC) (v1.0.5) (Lin and Peddada, 2020). The fold change between control samples and IBD samples (UC and CD) was determined and a Bonferroni multiple comparison correction was applied to the *p*-values.

## Results

### Overview of Studies Included in Dataset

In order to assess the cross-batch performance of each pipeline, we implemented a LODO cross validation approach. We collected 16S rRNA gene next generation sequencing data from 15 studies in North America for a total of 7,707 samples, comprising 55% healthy and 45% IBD samples, of which 56% are CD and 44% are UC (Table 2). The mean sequencing depth for each study ranged from 17547.5±7,522.5 to 402901.1±77178.6 reads per sample (Table 1). We included studies that contained IBD and control samples, only IBD samples, or only control samples in order to better recapitulate a diagnostic scenario, where any distribution of IBD and non-IBD samples may be received and processed together for disease classification. As some of the data processing methods share information across samples, it was important to test the pipeline’s performance on datasets with a range of label distributions that may be encountered.

**TABLE 2:** Overview of 15 datasets used to compare the effect of different features, data preprocessing methods, and machine learning models on IBD classification performance. Available metadata (age, sex, BMI, disease activity, and medication use) is provided for each dataset. Blank spaces indicate that the respective metadata was not available for the dataset’s samples.

Study	Accession	Disease Type	Number of Samples	Sample Type		Age		Sex			BMI		Disease Activity		Medications		
				Stool	Biopsy	Mean	SD	F	M	O	Mean	SD	Active	Rem-ission	Biologics	Immuno-suppresants	5-ASA
American Gut	PRJEB11419	Control	1,279	1,279	0	46.5	12.2	600	595	1	23.3	2.7	—	—	—	—	—
CVDF	PRJNA308319	Control	39	39	0	25.4	4.2	15	24	—	24.0	2.9	—	—	—	—	—
GEVERSC	—	CD	219	219	0	12.0	2.9	87	132	—	—	—	—	—	—	—	—
	PRJEB13680	Control	28	28	0	12.3	3.5	10	18	—	—	—	—	—	—	—	—
	—	UC	37	37	0	11.8	3.6	22	15	—	—	—	—	—	—	—	—
GEVERSM	—	CD	689	166	523	19.6	14.2	312	377	—	—	—	—	—	15	31	51
	PRJEB13679	Control	320	7	313	14.0	9.8	157	163	—	—	—	—	—	—	—	—
	—	UC	268	106	162	24.9	17.5	121	147	—	—	—	—	—	2	5	52
GLS	PRJEB23009	CD	340	340	0	30.2	9.0	215	102		25.7	7.2	43	297	145	74	15
		Control	335	335	0	48.6	14.4	152	166		32.8	8.4	—	—	—	—	—
HMP	—	CD	66	0	66	23.5	13.0	32	34	—	—	—	—	—	—	—	—
	ibdmdb.org	Control	43	0	43	28.7	22.0	20	23	—	—	—	—	—	—	—	—
	—	UC	36	0	36	27.7	17.4	20	16	—	—	—	—	—	—	—	—
MUC	PRJNA317429	CD	35	0	35	14.5	3.5	13	22	—	—	—	—	—	—	—	—
		Control	47	0	47	11.9	3.4	21	25	—	—	—	—	—	—	—	—
PRJNA418765	PRJNA418765	CD	589	589	0	40.4	13.2	332	257		26.4	6.6		589	416	—	—
PRJNA436359	PRJNA436359	UC	1,178	917	261	12.6	3.3	582	596	—	—	—	875	303	—	—	—
QIITA10184	PRJEB13895	Control	962	962	0	—	—	—	—	—	—	—	—	—	—	—	—
QIITA10342	PRJEB13619	Control	58	58	0	43.2	15.3	—	—	—	31.0	7.5	—	—	—	—	—
QIITA10567	PRJEB14674	Control	133	133	0	70.3	8.6	—	—	—	28.3	5.7	—	—	—	—	—
QIITA1448	PRJEB13051	Control	23	23	0	—	—	—	—	—	—	—	—	—	—	—	—
QIITA2202	PRJEB6518	Control	516	516	0	29.6	4.8	516	—	—	—	—	—	—	—	—	—
QIITA550	PRJEB19825	Control	467	467	0	32.8	0.5	131	336	—	—	—	—	—	—	—	—
**Total**	—	—	**7,707**	**6,221**	**1,486**	—	—	**3,358**	**3,048**	**1**	—	—	**918**	**1,189**	**578**	**110**	**118**

Bold values indicate the sum of the corresponding column.

### Overview of Leave-One-Dataset-Out Cross Validation

**FIGURE 1:**
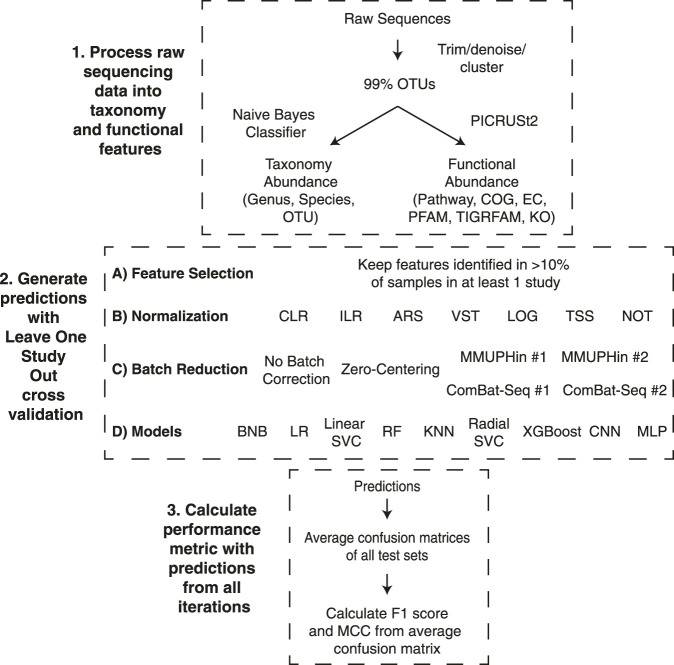
Leave-one-dataset-out cross-validation pipeline. The experiments comprised three different stages to go from raw sequence files to the performance metrics. 1) Raw sequences were processed with Dada2 or Deblur and close-reference clustered into OTUs at 99% identity. The OTUs were classified to taxonomy at 99% confidence with a Naive Bayes classifier and used to infer functional profiles with PICRUSt2. 2) Generating predictions for the 15 iterations of our LODO cross validation consisted of all possible combinations of the listed feature selection method, normalization or transformation methods, batch effect correction methods, and models. 3) The average confusion matrix proportions across each iteration was used to generate the overall confusion matrix. The F1 Score and MCC were calculated using the proportions from the average confusion matrix. The descriptions of acronyms and abbreviations are the following: Clusters of Orthologous Groups of proteins (COG), Kyoto Encyclopedia of Genes and Genomes (KEGG) orthologs (KO), Enzyme Commission (EC), Pfam protein domain (PFAM), TIGR protein family (TIGRFAM) and MetaCyc pathways (pathway), centered log-ratio (CLR), isometric log-ratio (ILR), arcsine square root transformation (ARS), variance stabilizing transformation (VST), log transformation (LOG), total sum scaling (TSS), no normalization (NOT), Bernoulli Naive Bayes (BNB), logistic regression (LR), linear support vector machine (Linear SVC), random forest (RF), K nearest neighbours (KNN), radial support vector machine (Radial SVC), eXtreme Gradient Boosting (XGBoost), convolutional neural network (CNN), multilayer perceptron (MLP).

In order to evaluate the different pipelines, we completed 15 cross-validation iterations with the classification model trained on n-1 datasets (all samples from a single dataset were removed) and the model performance assessed on the removed dataset (Figure 1). We evaluated the ability to classify samples from patients with IBD or non-IBD controls using different combinations of three taxonomic feature sets or six functional feature sets, six normalization methods, two transformation methods, six batch effect correction methods, and nine machine learning models (Figure 1). The binary classification performance of each combination of feature set, normalization or transformation, batch effect correction, and machine learning model was assessed with two classification metrics: F1 score and Matthews Correlation Coefficient (MCC) (Wardhani et al., 2019). In order to include datasets with a single label in the overall performance assessment, we calculated the overall classification performance from a confusion matrix comprising the average true positive, true negative, false positive, and false negative proportions across the 15 studies. Performance metrics are reported as median (25% percentile-75% percentile) of all pipelines containing the respective component. We performed a Mann-Whitney U test to determine if the performance was significantly different between different pipeline components.

### Non-Linear Models Achieve Greatest Classification Performance

Machine learning classification models identify decision boundaries within the feature space to separate one datapoint from another. For some ML models (BNB, Linear SVC, LR), these boundaries are linearly constrained, whereas others (RF, KNN, MLP, Radial SVC, XGBoost) can identify more complex, non-linear relationships between features and class. We assessed the generalizability of three linear and five non-linear ML models across the taxonomy and functional feature sets.

**FIGURE 2:**
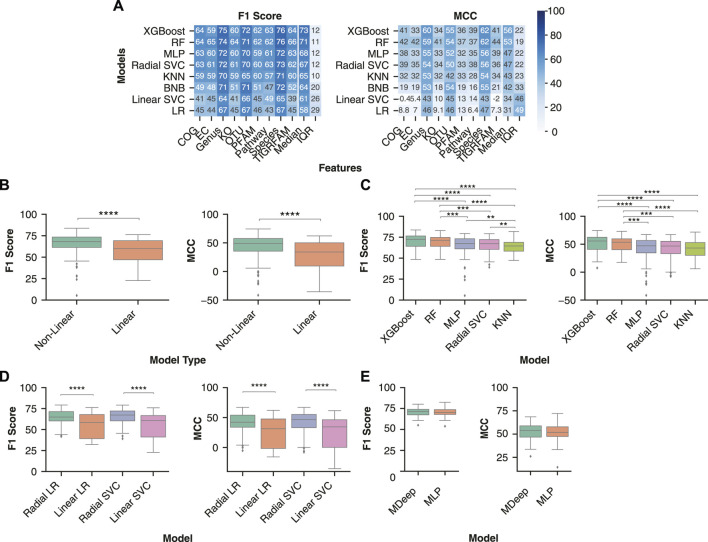
Non-linear models are better suited to identify decision boundaries between control and IBD samples than linear models. **(A)** Median model performance for each feature set across normalization, transformation, and batch effect correction methods. Rows were sorted in descending order by median performance across all feature sets. **(B)** Performance distribution of non-linear (RF, MLP, KNN, XGBoost, radial SVC) and linear (BNB, Linear SVC, LR) models. **(C)** Distribution of classification performance with the non-linear and linear variations of logistic regression and support vector machines across all feature sets. **(D)** Distribution of IBD classification performance between the non-linear models. The analysis comprised datasets preprocessed using all normalizations and transformations (ILR, CLR, VST, ARS, LOG, TSS, NOT) and batch effect correction (no batch effect correction, zero centering, MMUPHin #1, MMUPHin #2) methods performed on all feature types. **(E)** Comparison of two neural network architectures: the convolutional neural network MDeep or a MLP. A Mann-Whitney U test with Bonferroni correction was performed to compare all pairwise combinations of models with the significant comparisons indicated. ** indicates *p*-value < 0.01, *** indicates *p*-value < 0.001, **** indicates *p*-value < 0.0001.

The various ML models were sorted by the performance metrics’ median value across all pipelines containing the respective model. Across both performance metrics, the five models with the greatest classification performance were consistently the non-linear models, with the three linear models exhibiting the lowest classification performance (Figure 2A). The two models with the greatest median performance across were XGBoost, with a median F1 Score of 72.5 (64.1–76.4) and MCC of 55.6 (40.6–62.4) and Random Forest, with a median F1 Score of 71.2 (64.1–74.8) and MCC of 53.4 (40.2–59.6).

In addition, some combinations of linear models and functional features performed worse than randomly classifying the samples. For example, COG functional features with a logistic regression model had a median MCC of 8.8. Overall, non-linear models had significantly better classification performance than linear models, with a median F1 score [68.1 (61.4–73.5) vs 60.0 (47.1–69.2), *p*-value < 0.0001] and MCC [49.1 (35.5–57.7) vs 34.0 (9.6–50.1), *p*-value < 0.0001] (Figure 2B). Of the non-linear models, XGBoost and Random Forest had significantly higher F1 score and MCC than a MLP, KNN, or radial SVC (Figure 2C).

In order to further assess whether the non-linearity of a model improves classification in the context of microbiome data, we compared linear and non-linear variations of a support vector machine and logistic regression. Comparison of the two variations enables direct analysis of the impact of decision boundary constraints on performance, independent of differences in model architecture (Figure 2D). The non-linear (Radial) version of logistic regression had significantly greater F1 score (64.9 (60.3–71.4) vs 58.5 (39.2–67.9), *p*-value < 0.0001) and MCC [42.3 (33.8–53.9) vs 31.3 (1.6-47.7), *p*-value < 0.0001] than the linear (Linear) logistic regression. Additionally, the radial support vector machine had significantly greater F1 score [67.3 (60.4–72.2) vs 60.7 (41.1–66.7), *p*-value < 0.0001) and MCC (46.6 (33.1–55.2) vs 34.4 (0.2–46.3), *p*-value < 0.0001] than the linear version. In conclusion, non-linear models provided more accurate IBD classification, likely due to the complex relationships between features and disease labels.

Other ML model architectures, such as convolutional neural networks (CNNs), are commonly used for classification problems with defined structure in the input data, such as image classification. In the context of microbiome data, the CNN MDeep adds structure to OTU features through hierarchical agglomerative clustering of the phylogeny-induced correlation between OTUs (Wang et al., 2021). As MDeep is currently only developed for OTU features, we assessed whether this CNN architecture led to greater classification performance with OTU abundance than our MLP architecture. Comparison of each performance metric across all normalization, transformation, and batch effect correction methods indicated MDeep performance was not significantly different from our MLP model (MDeep F1 Score of 71.6 (67.5–74.0) vs MLP F1 Score of 70.3 (67.9–73.8), *p*-value > 0.05, and MDeep MCC Score of 54.0 (46.7–58.8) vs MLP F1 Score of 51.9 (47.4–57.9), *p*-value > 0.05) (Figure 2E).

### Top Inflammatory Bowel Disease Classification Was Obtained Using Taxonomic Features

Taxonomic features (species, genus or OTU) are predominantly used as input for ML models, whereas it is less common to use inferred functional features from PICRUSt2 as input. However, previous studies have identified lower inter-individual variation of the gut microbiome’s inferred functional profile than taxonomy (Davenport et al., 2014; Moustafa et al., 2018), suggesting that functional features may lead to better classification performance and generalizability. We processed the 16S sequencing samples with QIIME2 and PICRUSt2 to obtain taxonomy and functional feature abundance estimates, respectively.

**FIGURE 3:**
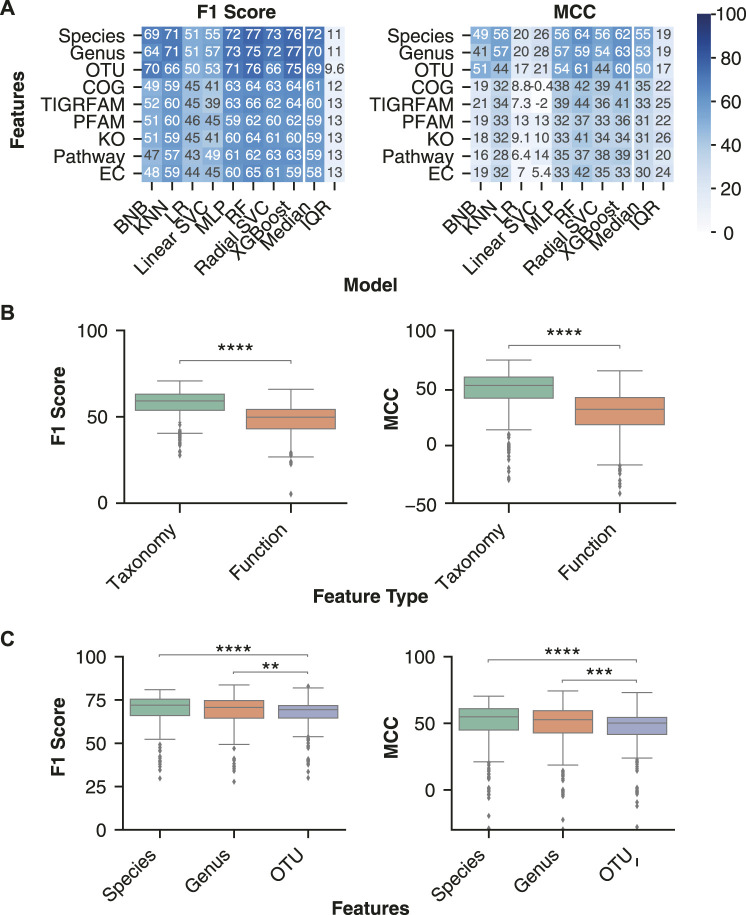
Optimal disease classification of microbiome samples obtained with taxonomic features. **(A)** Median performance of the three taxonomy and six functional feature sets for each ML model architecture. Rows were sorted in descending order by the mean column followed by the standard deviation (SD) column. **(B)** Distribution of performance metrics for taxonomy and functional features across all normalization or transformation, batch effect correction, and model combinations. **(C)** Distribution of classification performance with the three taxonomic feature sets. Independent Mann-Whitney U tests were performed to compare aggregate performance of taxonomy and functional features. The analysis was limited to all normalizations and transformations (ILR, CLR, VST, ARS, LOG, TSS, NOT) and batch effect correction (only no batch effect correction or zero centering) methods that were performed on all feature sets. ** indicates *p*-value < 0.01, *** indicates *p*-value < 0.001, **** indicates *p*-value < 0.0001.

For each ML model, we assessed the performance with taxonomy and functional abundance features in combination with a normalization or transformation and batch effect correction method. The batch effect correction methods were limited to Zero-Centering and no batch effect correction, as they were the only two performed on all datasets. Across all classification performance metrics, the taxonomic features classified IBD samples more effectively than functional features (Figure 3A). Comparison of performance with taxonomy and functional features confirmed the significantly higher F1 score [70.1 (63.8–74.7) vs 59.1 (51.2–64.3), *p*-value < 0.0001] and MCC [52.3 (41.2–59.5) vs 31.5 (18.1–41.7), *p*-value < 0.0001] for classification of IBD samples with taxonomic features (Figure 3B). Therefore, ML models using taxonomic features from this dataset lead to better classification of IBD samples than functional features.

We further investigated whether specific taxonomic ranks allow for an improved disease classification. Taxonomic classification consists of seven hierarchical ranks, with kingdom and species at the top and bottom, respectively. Each consecutively lower taxonomy rank provides greater resolution of the gut microbiome’s composition while also increasing data sparsity, which can negatively affect an ML model’s performance (Karlsson and Bostrom, 2014). Previous literature comparing different taxonomy ranks for disease classification indicated that lower ranks, down to genus, improved performance (Bang et al., 2019). We assessed whether the trend for improved classification continued with the species rank and OTUs, despite their increasing sparsity. While no significant performance difference was observed between species and genus ranks, both displayed significantly higher classification performance than OTU features (Figure 3C).

Due to the significantly better performance of non-linear classification models and taxonomic features, our subsequent analysis of normalization, transformations, and batch effect correction methods utilized only taxonomic feature sets and non-linear models.

### Evaluation of Normalization and Transformation Methods on Classification of Inflammatory Bowel Disease Samples

We assessed normalization and transformation methods which account for different biases commonly observed in next-generation sequencing data: compositionally, heteroskedasticity, and skewness. We selected two transformations designed for compositional data: the isometric log ratio (ILR) and centered log ratio (CLR) (Pawlowsky-Glahn and Egozcue, 2006). We selected two normalization methods which aim to reduce the heteroskedasticity: the arcsine square root (ARS) transformation (Campbell et al., 1970) of the total sum scaling (TSS) values and the variance stabilized transformation (VST) from the R package DESeq2 (Love, 2014). Next, we assessed a log transformation of the TSS values (LOG), which reduces the positive skew commonly seen in the distribution of microbiome data. Lastly, we assessed normalization by TSS alone to remove differences in sequencing depth between samples as well as the effects of not using any normalization.

**FIGURE 4:**
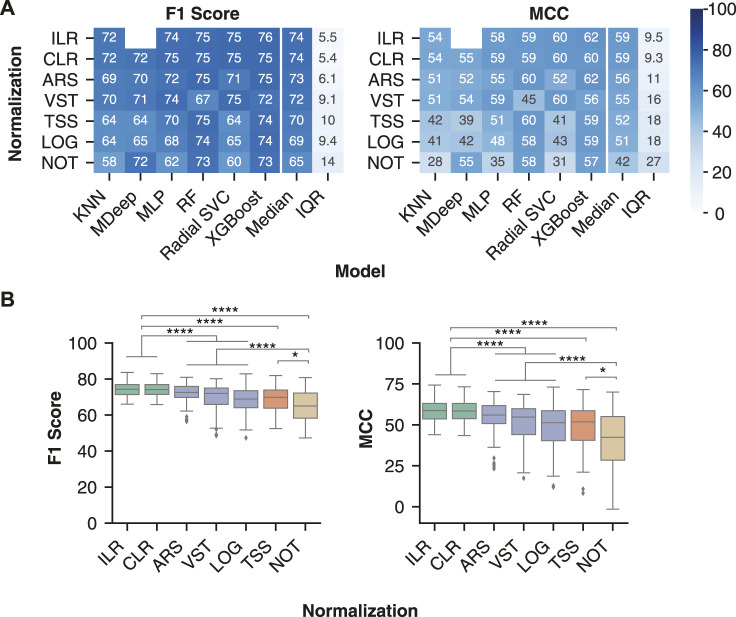
Compositional transformation methods lead to the highest model performance for IBD classification. **(A)** Median model performance with each normalization or transformation method across all batch effect correction methods. **(B)** Distribution of classification performance of different classes of normalization methods. The compositional category consists of CLR and ILR (green), variance/distribution modifiers consist of VST, ARS, and LOG(blue), scaling consists of TSS (orange), and no normalization consists of NOT (brown). Classification performance following data processing with all pairwise combinations of the normalization or transformation methods (ILR, CLR, LOG, ARS, VST, TSS and NOT) and batch effect correction methods (No batch effect correction, MMUPHin #1, MMUPHin #2, ComBat-seq #1, ComBat-seq #2, and Zero-Centering) were included. Rows were sorted in descending order by the median of each performance metric across the non-linear models. No analysis was performed for MDeep paired with ILR as the ILR normalized values no longer map directly to a feature, therefore removing the phylogenetic structure required for MDeep. **** indicates *p*-value < 0.0001 and * indicates *p*-value < 0.05.

The compositional transformation methods were the most generalizable across non-linear models (Figure 4A), with median ILR F1 score of 74.3 (71.4–76.9) and MCC of 58.7 (53.6–63.1) and median CLR F1 score 74.2 (71.5–76.9) and MCC 58.5 (53.8–63.1). The compositional transformations were followed by the variance/distribution modifiers ARS (F1 score of 72.5 (69.8–75.8) and MCC of 56.0 (50.8–61.8)) and VST (F1 score 72.0 (65.9–75.0) and MCC 54.8 (44.1–59.9)). Lastly, TSS [F1 score 69.8 (63.8–73.9) and MCC 51.9 (40.5–58.7)] and LOG[F1 score 68.9 (64.1–73.5) and MCC 51.3 (40.4–58.6)] were consistently the lowest performing normalization. Furthermore, the compositional methods led to significantly better F1 score and MCC than the other normalization type (Figure 4B), whereas the variance/distribution modifiers and scaling method were significantly better than no normalization. These results indicate the importance of transformation methods which account for the compositional properties of microbiome data prior to model training.

### Evaluation of Batch Effect Correction Methods on Classification of Inflammatory Bowel Disease Samples

Various approaches have been proposed to remove technical batch effects from next generation sequencing datasets, of which we selected three relevant to microbiome data. First, the zero-centering method aims to reduce batch effects by centering the mean of each feature within a batch to zero (Nygaard et al., 2016). Second, Meta-analysis Methods with a Uniform Pipeline for Heterogeneity in microbiome studies (MMUPHin) (Ma et al., 2020) implements an empirical Bayes’ approach to estimate and remove batch-specific parameters for each feature. Whereas, ComBat-seq implements a negative binomial regression model to estimate and correct batch effect parameters (Zhang et al., 2020).

**FIGURE 5:**
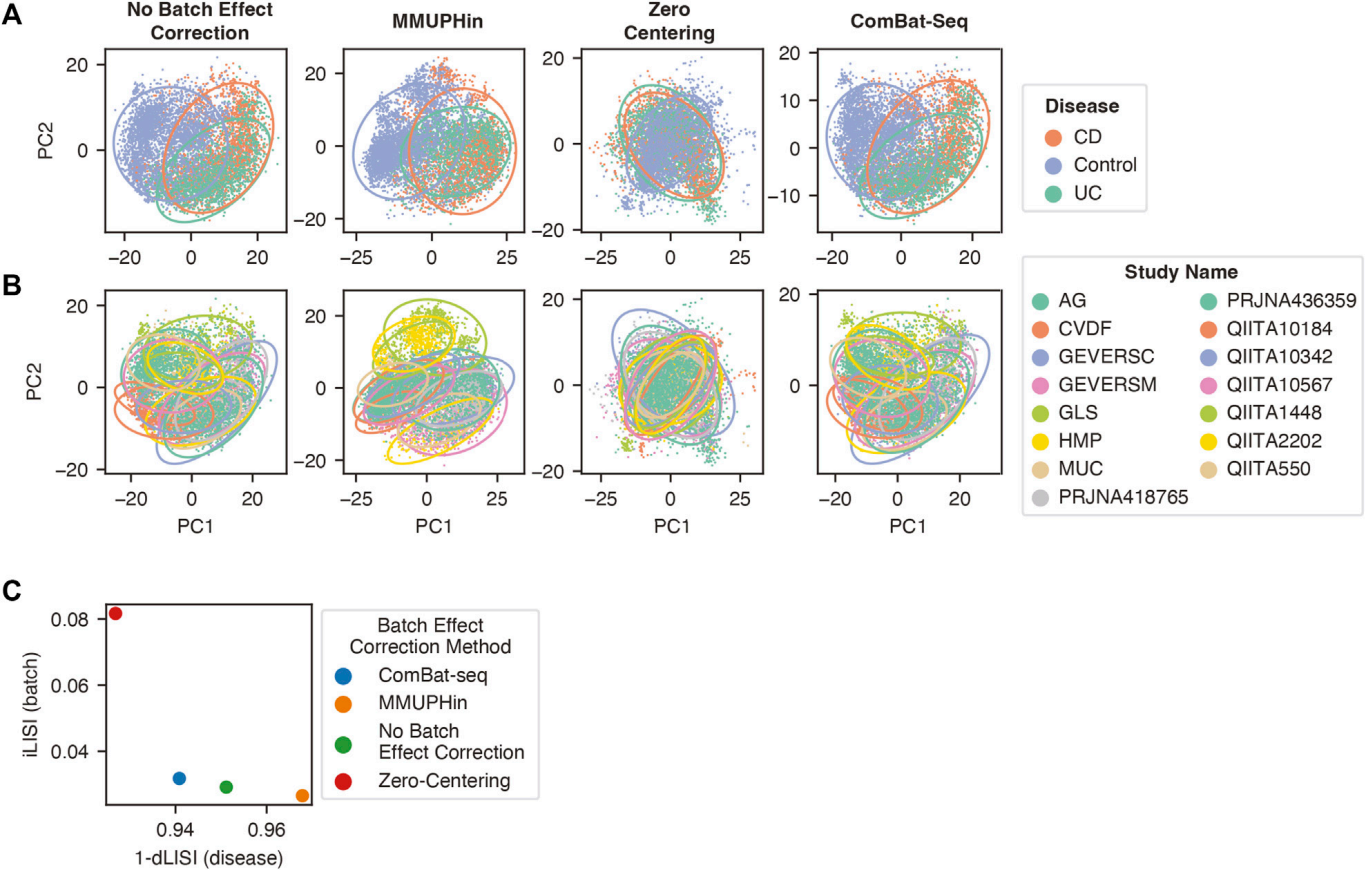
Batch effect correction by zero-centering, MMUPHin, and ComBat-seq. Principal component analysis of genus features follow CLR transformation and batch effect correction with the points coloured by disease label **(A)** or study label **(B)**. The 95% confidence ellipse for each category within the label is shown to better visualize the mixing and separation of different labels. **(C)** The normalized median LISI value for the batch label (*y*-axis) and the 1-normalized median LISI value for the disease label (*x*-axis).

We first compared the ability of these three methods to correct batch effects in our dataset. MMUPHin and ComBat-seq were provided the disease and sample type as biological covariates alongside the batch label, whereas zero-centering was blind to biological covariates and only provided the batch label. The performance was evaluated with the beta-diversity Aitchison distance between samples (Figure 5A,B) (Aitchison, 1982) and the Local Inverse Simpson Index (LISI) (Figure 5C) (Polański et al., 2020). LISI evaluates the local neighborhood of a sample with respect to the batch (iLISI) or disease label (dLISI). A higher value indicates the presence of samples with a greater variety of labels in the surrounding neighborhood. A good batch effect correction method will lead to an increased iLISI (improved mixing of batches) and a decreased dLISI (improved separation of disease labels).

Compared to no batch effect correction, MMUPHin improved separation of disease labels (1-dLISI of 0.968 vs 0.951, *p* < 0.0001), whereas it led to greater separation of batches in our dataset (iLISI of 0.027 vs 0.029, *p* < 0.0001) (Figure 5A–C). Conversely, ComBat-seq reduced separation of disease labels (1-dLISI of 0.941 vs 0.952, *p* < 0.0001) and improved mixing of batches (iLISI of 0.032 vs 0.029, *p* < 0.0001) compared to no batch effect correction (Figure 5A–C). The naive zero-centering method led to the greatest mixing of disease labels (1-dLISI of 0.927 vs 0.951, *p* < 0.0001) and greatest mixing of batches (iLISI of 0.082 vs 0.029, *p* < 0.0001) compared to no batch effect correction (Figure 5A–C). For the combined assessment of the dLISI and iLISI scores, we calculated the harmonic mean (F1 score) (Lin et al., 2019; Tran et al., 2020), where a higher mean indicates a better batch effect correction method. The F1 score indicated the performance order of batch effect correction methods was naive zero-centering (0.150), ComBat-seq (0.061), and MMUPHin (0.052), which was lower than no batch effect correction (0.057).

### Evaluation of Batch Effect Reduction Methods on Classification of Inflammatory Bowel Disease Samples

We assessed the effect of three batch effect correction methods on the classification performance of non-linear models. Two aspects of MMUPHin and ComBat-seq were accounted for to ensure a similar scenario of an implemented diagnostic test. First, the disease covariate is commonly provided alongside the batch label, which is unknown for a diagnostic test. Second, information is shared across batches in order to correct the batch effect estimates. For a diagnostic test, new test datasets would be continually received and have to undergo batch effect correction independent of the training dataset. Therefore, we implemented two variations to simulate the diagnostic scenario of obtaining a new dataset with unknown disease labels. The first (#1) method applied batch effect correction to the training and test sets separately providing the sample type (stool/biopsy) as the biological covariate. Whereas the second (#2) method only applied batch effect correction to the training set with the disease and sample type covariates provided (see Methods for detailed description). On the other hand, batches are independent in the zero-centering method allowing it to be implemented without the training and test sets affecting each other.

**FIGURE 6:**
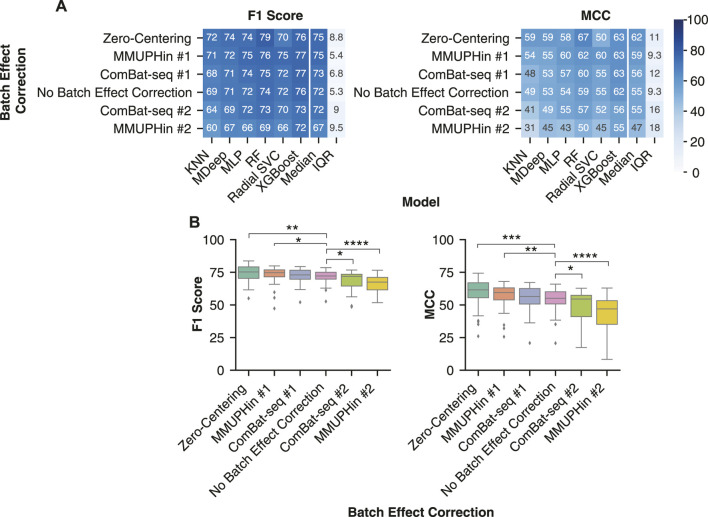
Removing batch effects with zero-centering improved IBD classification. **(A)** Median performance of each batch effect correction method across all combinations of normalization or transformation methods, taxonomic features, and non-linear ML models. Rows were sorted in descending order by the median performance across all non-linear models. **(B)** Distribution of IBD classification performance following batch effect correction by the six different methods. All batch effect corrections were compared with a Mann-Whitney U test to the No Batch Effect Correction performance and the significant comparisons were labelled. * indicates *p*-value < 0.05, ** indicates *p*-value < 0.01, *** indicates *p*-value < 0.001, and **** indicates *p*-value < 0.0001.

Of the six different batch effect correction variations, zero-centering and MMUPHin #1 were the most generalizable approach across the non-linear models. Zero-centering led to a significantly greater F1 score of [75.3 (70.3–79.2) vs 72.2 (69.8–75.0), *p* < 0.01] and MCC [61.6 (55.6–67.0) vs 55.1 (50.8–60.1), *p* < 0.001] than no batch correction (Figure 6A,B). MMUPHin #1 also led to a significantly greater F1 score of [74.7 (71.5–76.9) vs 72.2 (69.8–75.0), *p* < 0.05] and MCC [59.4 (53.9–63.1) vs 55.1 (50.8–60.1), *p* < 0.01] than no batch correction (Figure 6A,B). Whereas, ComBat-seq #1 displayed greater median F1 scores of 71.9 (64.5–73.5) and MCC of 54.5 (41.0–57.4) than no batch reduction, although not significant. Classification performance with MMUPHin #1 and ComBat-seq #1 was more consistent across the different non-linear models than zero-centering, likely due to the poor performance of the radial SVC with Zero-Centering. (Figure 6A). Whereas, MMUPHin #2 and ComBat-seq #2 were the least generalizable (Figure 6A) with significantly lower F1 score of 67.5 (61.5–71.1) (*p* < 0.0001) and 71.9 (64.5–73.5) (*p* < 0.05) and MCC of 47.0 (35.2–53.3) (*p* < 0.0001) and 54.5 (41.0–57.4) (*p* < 0.05) than no batch effect correction MCC of 54.6 (47.7–59.8), respectively. Therefore, the naive zero-centering MMUPHin #1 method for batch effect correction are the most generalizable approach for IBD classification with non-linear machine learning models.

### Evaluation of Model Performance on Sample and Patient Subgroups

The samples used to assess the performance of different combinations of normalizations or transformations, batch effect correction, and ML models were drawn from across sample collection methods (i.e., stool and biopsy) and patient demographics (i.e., paediatric and adult samples). While we did not set inclusion criteria for samples based on these differences, previous research has demonstrated distinct differences in microbiome composition between sample types and demographic groups (Walters et al., 2014; Kim et al., 2020; Radjabzadeh et al., 2020). For example, principal coordinate analysis (PCoA) with weighted UniFrac distance of (Durbán et al., 2011) and principal component analysis (PCA) of CLR-transformed taxonomic features indicated paired biopsy and stool samples from the same individual cluster separately (Mas-Lloret et al., 2020).

We compared the model performance for the sample and patient demographics for which we were able to acquire sufficient metadata and have been associated with microbiome alterations: sample type (biopsy vs stool), IBD type (CD vs UC), sex (Female vs Male), BMI (BMI <30 vs BMI >30), and age (Adult vs Pediatric). To assess the performance within each demographic, we included the predictions from taxonomic features (species, genus, OTU) with a compositional transformation method, zero-centering batch effect correction, and a non-linear ML model. Our analysis focused on the MCC performance metric as it is more robust to imbalanced label distribution (Chicco and Jurman, 2020), which occurred when the samples were grouped by the five metadata categories. A logistic regression function was used to assess changes in performance corresponding to each demographic while controlling for the other metadata (Table 3).

**TABLE 3:** Model performance for different sample types and patient demographics. Samples with available metadata were categorized into groups based on the collection method or the patient’s specific demographic group based on sex, age, and BMI. Predictive performance for all combinations of taxonomic features, compositional transformations, zero-centering batch effect correction, and non-linear models were included in the analysis. Logistic regression was performed to assess the performance differences within each sample and demographic group while adjusting for the remaining covariates. **** indicates *p*-value < 0.0001, and * indicates *p*-value < 0.05.

Group	Variable	Coefficient	SE
Sample Type	Biopsy (vs Stool)	−0.44 *	0.2
Life Stage	Adult (vs Pediatric)	1.39 ****	0.18
BMI Stratification	BMI <30 (vs BMI >30)	−0.85 ****	0.19
Sex	Female (vs Male	−0.02	0.18
IBD Type	CD (vs UC)	0.05	0.18

IBD classification performance was reduced for biopsy samples compared to stool samples, increased for samples from adult patients compared to pediatric patients, and decreased for samples from patients with BMI less than 30 compared to patients with BMI greater than 30. On the other hand, there was no difference in classification performance for females compared to males or for samples from patients with CD compared to patients with UC (Table 3). The metadata groups with different performance between the two categories coincided with those that are not equally represented in our dataset, highlighting the importance of accounting for different demographic groups in a microbiome based diagnostic test.

### Evaluation of Top Performing Pipeline Combinations for Inflammatory Bowel Disease Classification

Our analysis identified the features, ML models, normalization or transformation methods, and batch effect correction methods which led to the most generalizable performance. In order to determine the best overall combination of features, data processing, and ML model we assessed the top three performing models (Table 4). The top two models consisted of the most generalizable individual components: taxonomic features (genus), non-linear model (XGBoost), compositional transformation (ILR or CLR), and zero-centering to remove batch effects. In addition, the third best model consisted of the lowest performing taxonomic features (OTU) and the lowest performing normalization (LOG), with zero-centering for batch effect correction. Overall, the combination of the most individual most generalizable methods led to the top two pipelines with the best classification performance.

**TABLE 4:** Top three data processing and model pipelines for classifying IBD samples. Three combinations which appeared when all pipelines were sorted by F1 score or MCC.

Features	Normalizations	Batch Effect Correction	Model	F1 Score	MCC
Genus	ILR	Zero-Centering	XGBoost	83.7	74.3
Genus	CLR	Zero-Centering	XGBoost	83.0	73.2
OTU	LOG	Zero-Centering	Random Forest	82.9	73.1

**FIGURE 7:**
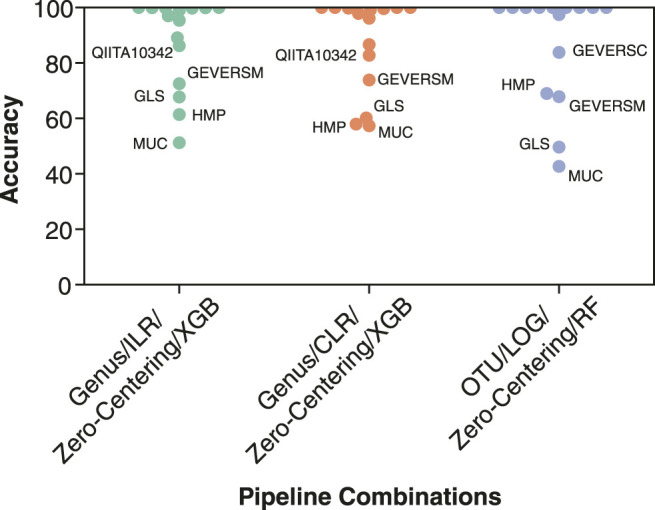
Individual classification accuracy of the 15 datasets by the top three performing pipeline combinations. Classification accuracy was calculated individually for each of the datasets from the 15 LODO iterations. The three pipelines consist of (1) genus features, ILR transformation, Zero-Centering batch effect reduction, and a XGBoost classifier (Genus/ILR/Zero-Centering/XGB), (2) genus features, CLR transformation, Zero-Centering batch effect reduction, and a XGBoost classifier (Genus/CLR/Zero-Centering/XGB), and (3) OTU features, LOG normalization, Zero-Centering batch effect reduction, and a Random Forest classifier (OTU/LOG/Zero-Centering/RF). Points corresponding to one of the five datasets with the lowest classification accuracy are labelled.

For the three best pipeline combinations (Table 4), we assessed the classification performance on each dataset from the fifteen LODO iterations. Since some studies contained only a single label, either non-IBD, control or IBD, we assessed the performance using the classification accuracy metric (Figure 7). The three pipeline combinations had the lowest performance on a common subset of the fifteen datasets. The low performance datasets were enriched in samples from sample types and patient subgroups that we previously showed negatively affect classification performance (Table 3), such as biopsy samples (MUC, HMP, and GEVERSM), patients with BMI >30 (GLS or QIITA10342), or pediatric patients (GEVERSC). These results further highlight the importance of generating a diverse training set that is representative of the patient demographics the diagnostic model will encounter.

### Identification of Important Features for Classification With a XGBoost Model

**FIGURE 8:**
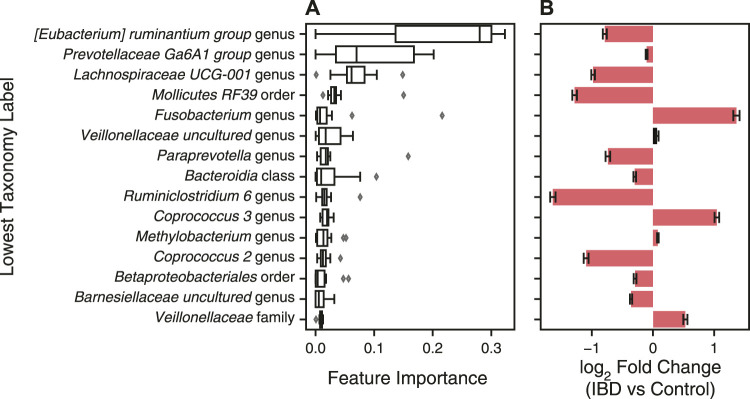
Features with greatest contribution to IBD classification with XGBoost classifier. **(A)** A XGBoost classifier was trained with CLR normalized genus abundance features with zero-centered batch effect correction for fifteen LODO iterations. The features’ gain values for each iteration were extracted and sorted by the mean gain across all iterations. Error bars represent mean ± standard error of the mean for the fifteen iterations. The lowest classification rank for each feature was used as the label for the corresponding bar. **(B)** Changes in taxonomy abundance between control samples and those from patients with IBD. Bars represent the fold change ± the standard error determined with Analysis of Compositions of Microbiomes with Bias Correction (ANCOM-BC). Red indicates a significant fold change between IBD and control samples (*p* < 0.05) and black indicates non-significant fold change.

In addition to predicting health diagnoses, machine learning models can be used to identify biomarkers for disease by identifying features important for disease classification. We characterized the feature importance from the second-best overall data processing and ML model pipeline (Table 4). We did not analyze the feature importance of the best-performing model because the ILR normalized values no longer correspond to the input features thereby preventing interpretation of feature importance. For an XGBoost model, the importance corresponds to a feature’s contribution to the model’s decision during training, referred to as the gain value (Chen and Guestrin, 2016). We extracted the features’ gain values from each of the 15 LODO iterations, sorted by the mean of all iterations, and plotted the top fifteen features (Figure 8). In addition, we determined the change in abundance for each taxonomy to assess whether our dataset aligned with previous findings on changes of the microbiome in IBD.

Many of most important taxa are in the short chain fatty acid (SCFA) producing Clostridium XIVa/IV clusters, including bacteria from the *Eubacterium*, *Coprococcus*, *Lachnospira*, and *Ruminiclostridium* genera (Figure 8A). Aligning with previous studies, these bacteria were decreased, with the exception of *Coprococcus 3*, in IBD samples vs control samples in our dataset (Figure 8B) (Gevers et al., 2014; Nagao-Kitamoto and Kamada, 2017). *Fusobacterium* and *Veillonellaceae* genera, commonly increased in the gut microbiome of IBD patients, were also top contributors to the XGBoost classifier (Figure 8A,B) (Gevers et al., 2014; Glassner et al., 2020). In addition, the *Prevotellaceae* genus was the second most important feature, with the decreased abundance in IBD samples agreeing with previous studies showing decreased abundance in the gut microbiome of patients with CD and UC (Figure 8A,B) (Chen et al., 2014). Even though some taxa have a low fold change between IBD and non-IBD controls, XGBoost is able to find a cutoff value that contributes to the separation of labels and the final decision. XGBoost classifiers have the best potential for use as a diagnostic test due to their performance as well as their interpretability and utility in identifying disease biomarkers.

## Discussion

We assessed how different feature sets, ML models, normalization or transformation methods, and batch effect correction methods affect predictive performance across patient cohorts in a LODO cross validation approach. The limited applicability of a PCR-based diagnostic test with a handful of bacteria for IBD diagnosis (Wyatt and Kellermayer, 2018) has led the field to explore the use of ML models for disease diagnosis. Our benchmark provides practical suggestions for ways to improve the performance of an IBD diagnostic test using the gut microbiome composition. First, genus abundance estimates from 16S rRNA sequencing need to be normalized by a compositional transformation method, with CLR transformation being the most appropriate as it allows for each feature’s importance to the ML models decision to be assessed. Second, zero-centering batch effect correction should be applied to each batch of samples collected, sequenced, and processed together to reduce systematic batch differences. Following normalization and batch effect correction, an XGBoost or random forest classification model should be trained and optimal hyperparameters determined through grid search and LODO cross validation for implementation as a diagnostic test. With respect to the training dataset, it is important to account for patient demographics or technical differences between samples that have been associated with gut microbiome alterations. We suggest several options for optimal performance: 1) ensure balanced representation in the training dataset, 2) include the metadata labels as a feature for the model, or 3) deploy diagnostic ML models built specifically for one demographic group. In addition, the LODO cross-validation methodology is an important tool for the selection of other, new data preprocessing and model building methods.

Previous studies have demonstrated greater consistency of functional feature abundances than taxonomic feature abundance in both healthy individuals (Turnbaugh et al., 2009; Human Microbiome Project Consortium, 2012; Zhernakova et al., 2016) and those with IBD (Davenport et al., 2014; Zhou et al., 2018). In fact, some studies were unable to identify a single bacteria present in every IBD patient from their cohort (Moustafa et al., 2018). The reduced variation and sparsity of functional features led us to hypothesize that functional abundance profiles would lead to better classification of IBD samples. However, through our LODO cross validation, we found that classification performance with functional features was significantly worse than with taxonomic features (Figure 3B). We postulate the reason for the reduced classification performance with functional profiles is due to the limited recapitulation of functional profiles with PICRUSt2 (Douglas et al., 2020; Sun et al., 2020) and the inability of 16S rRNA sequencing to identify strain-level functional differences of the present bacteria (Filippis et al., 2020). To overcome these limitations in future studies, measurement of the microbiome’s gene content by WGS, transcriptomes by RNA-seq, or metabolites by metabolomics need to be explored. In fact, functional profiles from whole genome sequencing led to better predictions of patients with IBD who achieved remission with vedolizumab than taxonomy abundance (Ananthakrishnan et al., 2017; Filippis et al., 2020). While whole genome sequencing may improve disease classification, its much higher cost than 16S rRNA sequencing substantially hinders the technology’s adoption as a diagnostic test.

A major hurdle in the implementation of sequencing based diagnostic tests in the clinic is the observed systematic differences between sample preparations. In a previous study, removal of these batch effects with an empirical Bayes’ or zero centering approach led to improved classification (Luo et al., 2010). We demonstrate similar results with significantly improved cross-batch classification performance with zero-centering and the empirical Bayes’ method MMUPHin (Figure 6). Of the two methods, zero-centering had slightly higher F1 Score and MCC and the top performing pipelines comprised zero-center batch effect reduction exclusively. In addition, the updated ComBat-seq did not significantly improve classification performance either. Aligning with our classification results, our assessment of the correction of batch effects with the LISI metric indicated the best batch effect correction method was zero-centering, whereas MMUPHin and ComBat-seq were more similar to no batch effect removal (Figure 5). Both MMUPHin and ComBat-seq are designed and optimized for disease mechanism and biomarker discovery where the disease covariate is known and incorporated into the method. The inclusion of a disease covariate is not applicable to a diagnostic scenario though, where the diagnosis label is to be determined, resulting in the need for a covariate agnostic method such as zero-centering. The lower improvement in classification performance with MMUPHin #1 or ComBat-seq #1 compared to no batch effect correction is potentially due to its implementation in a scenario the method was not optimized for.

Similar to batches of samples collected for a diagnostic test, the batches in our dataset were not balanced, with some containing only a single diagnosis class (e.g., all samples coming from IBD patients). In cases where the batch and diagnosis label are confounded, batch effect correction methods tend to reduce the disease associated differences in the process of removing the batch differences (Nygaard et al., 2016). Therefore, the more advanced removal of batch effects by MMUPHin and ComBat-seq likely led to an over-adjustment within the unbalanced batches and removal of the disease differences. Whereas, the simpler removal of batch effects with the covariate naive zero-centering approach retained sufficient biological signal between disease labels for non-linear ML models to correctly classify samples across batches. Batch effect correction methods that do not require input of a covariate have been developed, such as frozen surrogate variable analysis or reference principal component integration (RPCI) (Parker et al., 2014; Liu et al., 2021), although their applicability to microbiome data has not been assessed.

Although our study demonstrated reliable results, gaps in the publicly available data prevented us from several critical analyses. First, the identification of CD and UC patients relied on the accuracy of the diagnosis coding in the public databases. However, there were no studies explicitly validating the registration of CD and UC diagnosis codes. In addition, we lacked information on the timing of sample collection in relation to patients’ diagnosis and disease progression, current disease activity quantification, DNA extraction, and sample storage information. Furthermore, there was limited information on environmental factors such as medication usage, alcohol usage, smoking, diet, and other factors known to alter the gut microbiome which could affect our analysis (Zhou et al., 2018; Bryrup et al., 2019). Of the sample information and patient demographic data we did obtain, clear differences in performance of our top pipelines were observed between patient subpopulations. (Table 3)**.** Therefore, future studies with improved lifestyle and clinical metadata are needed to systematically address how these factors affect performance of a gut microbiome diagnostic test.

Other non-invasive diagnostic tests for IBD, such as fecal calprotectin, continue to have significant differences between the reports on the sensitivity and specificity for classifying IBD patients from non-IBD (Lewis, 2011). While high performance levels have been reported, one recent study identified a 78% accuracy for identifying patients with IBD using fecal calprotectin (Penna et al., 2020), which is approximately 10% lower than our best model. Furthermore, while we focused solely on IBD classification here, ML models using microbiome composition have wider applicability than singular biomarkers such as calprotectin. Models using high-dimensional microbiome data have already been developed to predict if a patient with IBD will respond to a medication (Ananthakrishnan et al., 2017), to predict a patient’s postprandial glycemic response (Zeevi et al., 2015), and classification of other diseases, such as Parkinson’s disease (Zeevi et al., 2015; Hill-Burns et al., 2017), to name a few.

With sufficient data and validation, analysis of the fecal gut microbiome can indeed be leveraged as a multi-purpose predictive tool. Given the significant delay (Vavricka et al., 2012; Zaharie et al., 2016; Nguyen et al., 2017) and associated costs of diagnosis (Park et al., 2020; Vadstrup et al., 2020), it is critical to continue exploration of approaches that increase accessibility of diagnosis and decrease the cost of testing (Zhang et al., 2019) in a community health or primary care setting. ML models with microbiome data have the potential to achieve these goals and further work to gather more well-annotated data, improve performance and assess models with validation studies is required.

## Data Availability

Publicly available datasets were analyzed in this study. This data can be found here: American Gut (PRJEB11419), CVDF (PRJNA308319), GEVERSC (PRJEB13680), GEVERSM (PRJEB13679), GLS (PRJEB23009), MUC (PRJNA317429), PRJNA418765, PRJNA436359, QIITA10184 (PRJEB13895), QIITA10342 (PRJEB13619), QIITA10567 (PRJEB14674), QIITA1448 (PRJEB13051), QIITA2202 (PRJEB6518), QIITA550 (PRJEB19825). The raw sequencing data for the HMP 16S rRNA dataset was downloaded from ibdmdb. org.
